# Observation of Morphology Changes of Fine Eutectic Si Phase in Al-10%Si Cast Alloy during Heat Treatment by Synchrotron Radiation Nanotomography

**DOI:** 10.3390/ma11081308

**Published:** 2018-07-28

**Authors:** Shougo Furuta, Masakazu Kobayashi, Kentaro Uesugi, Akihisa Takeuchi, Tomoya Aoba, Hiromi Miura

**Affiliations:** 1 Department of Mechanical Engineering, Toyohashi University of Technology, Toyohashi 441-8580, Japan; furuta@str.me.tut.ac.jp (S.F.); aoba@me.tut.ac.jp (T.A.); miura@me.tut.ac.jp (H.M.); 2 Research & Utilization Division, Japan Synchrotron Radiation Research Institute, Hyogo 679-5198, Japan; ueken@spring8.or.jp (K.U.); take@spring8.or.jp (A.T.)

**Keywords:** particle morphology, heat treatment, aluminum cast alloy, mechanical properties, Ostwald ripening, nanotomography, phase-contrast imaging

## Abstract

A series of three-dimensional morphology changes of fine eutectic Si-particles during heat treatment have been investigated in Self-modified and Sr-modified Al-10%Si cast alloys by means of synchrotron radiation nanotomography utilizing a Fresnel zone plate and a Zernike phase plate in this study. The coral-like shape particles observed in Sr-modified cast alloy fragmented at branch and neck during heat treatment at 773 K. The fragmentation occurred up to 900 s. After that, the fragmented particles grew and spheroidized by Ostwald ripening. On the other hand, rod-like shaped eutectic Si-particles observed in self-modified cast alloy were larger in size compared with the particle size in Sr-modified cast alloy. Separation of eutectic Si-particles in Self-modified cast alloy occurred up to approximately 900 s, which was similar tendency to that in Sr-modified cast alloy. However, it was found that the morphology change behavior was very complex in rod-like shape Si-particles. The three-dimensional morphology changes of fine eutectic Si-particles in both cast alloys, specifically fragmentation and spheroidizing, can be connected to changes in mechanical properties.

## 1. Introduction

Since the building of large synchrotron radiation facilities throughout the world in the latter half of the twentieth century, the performance of synchrotron radiation tomography has gradually improved up to the present day [1,2,3]. Currently, in the Japanese synchrotron radiation facilities, SPring-8, three-dimensional non-distractive observation with a spatial resolution of 50–160 nm is available constantly in the imaging beamline by using an X-ray focusing device of a Fresnel zone plate [4,5,6]. Phase-contrast imaging techniques have been also developed for those samples for which visualizations are difficult by X-ray absorption contrast (i.e., these densities are very close) [7]. Furthermore, improvement of the performance of X-ray 2D detector system has rapidly shortened scanning time. Therefore, synchrotron radiation tomography can be used for various studies in various fields.

The advantages of X-ray tomography are that three-dimensional morphologies are obtained, and that the observation is non-destructive. In studies of structural materials, material behaviors changing over time can be visualized, for instance, damage and fracture mechanisms [7,8,9,10,11,12,13,14], fatigue and crack propagation phenomena [15,16,17,18] and so on. We can deeply understand various phenomena affected by microstructures from a series of observed images. In Al-Si cast alloys, it is well known that eutectic Si-particle strongly affects mechanical properties. Many studies with regard to the morphology and distribution of eutectic Si-particles have been conducted to date [19,20,21], because the spheroidizing of Si-particles brings, in particular, ductility improvement by heat treatment. However, most of the research had been performed on the basis of 2D observation by polishing of a heat-treated sample after cross section cutting. Although three-dimensional evaluation also exists using Focus Ion Beam tomography [22], unfortunately this method is destructive.

In the application of hypoeutectic Al–Si alloys for automobile parts which require sufficient toughness, Si-particle refinement is applied by adding trace Sr to improve the mechanical properties [23,24,25]. The addition of trace Sr prevents aluminum phosphide, AlP which become the nuclei of coarse Si particles [26], and then changes the solidification process of hypoeutectic Al-Si alloys [27]. Note that the origin of phosphorus is the impurity of Si. Si-particles modified by trace Sr addition become very fine at less than 1 μm. A Sr-modified hypoeutectic Al-Si alloy demonstrates excellent mechanical properties. Furthermore, with applying heat treatment to the alloy, its strength and ductility can be controlled. The changes of Si-particles morphology during a heat treatment are considered as follows; firstly, Si-particles with necking divide into parts by Plateau–Rayleigh instability [28,29]. This separation is a change which decreases system energy quickly. Next, the fragmented Si-particles grow into spherical shapes by diffusion-controlled Ostwald ripening to reduce their surface energy.

By contrast, self-modification (self-refinement) of eutectic Si-particles is also possible by killing an impurity element of P, which is contained in Si and forms AlP as the solidification nuclei of Si. This P-free solidification process has been reproduced by phase-field model simulation by Eiken [27]. The morphologies of eutectic Si-particles which are formed in the different solidification processes—self-modification and Sr-modification—are different. Synchrotron radiation nanotomography has revealed that the morphologies of Si-particles in self-modification and Sr-modification are of a rod-like shape and coral-like shape, respectively, by casting self-modified and Sr-modified samples and investigating practically [30]. Therefore, in this study, to clarify the behavior of morphological changes during heat treatment and the effect of them on mechanical properties, hypoeutectic Al-10%Si alloys were cast using two different solidification processes (Self-modification and Sr-modification) that produce different morphology of eutectic Si-particles (rod-like and coral-like). The changes of mechanical properties were investigated in the prepared samples. The three-dimensional morphology changes of eutectic Si-particles during the heat treatment process were observed in both alloys by using nanotomography with a Fresnel zone plate and a Zernike phase plate.

## 2. Materials and Methods

**Figure 1:**
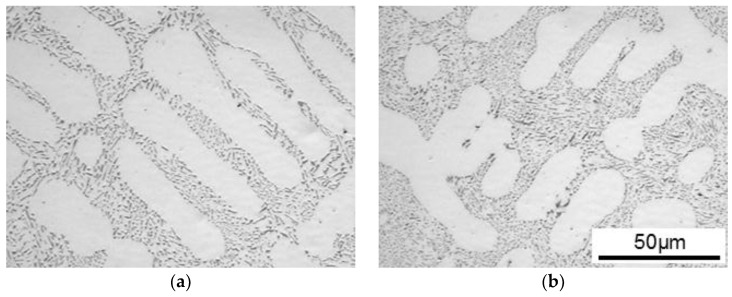
Optical micrographs; (**a**) Al-9.8%Si-3ppmP cast alloy and (**b**) Al-10.1%Si-4ppmP-108ppmSr cast alloy.

Al-10%Si alloy was selected as the sample of this study. Two kinds of Al-10%Si alloy, self-modified and a Sr-modified sample, were prepared by gravity casting. It is known that three-dimensional morphology of eutectic Si-particles is different between the two alloys [30]. High purity Al (99.99%) and high purity Si (99.9999%) were melted in a graphite crucible at 993 K in air atmosphere using an electrical resistance furnace (Hamamatsu heat-tech, Hamatsu, Shizuoka, Japan). The molten metal was degassed by hexachloroethane. After the degassing treatment, molten metal was cast into a boat-shaped iron-mold heated at 473 K with a cavity size of 150 mm × 25 mm × 25 mm as a self-modified sample. For the Sr-modified sample, the degassed molten metal was cast into the mold soon after the 100 ppm Sr addition. The chemical compositions of cast alloy samples detected by spark emission spectrometer (OBLF QSN750-II, Witten, Germany) are listed in Table 1. Hereafter, two prepared cast alloys are named as Al-9.8%Si-3ppmP and Al-10.1%Si-4ppmP-108ppmSr on the basis of the result of composition analysis. Photos of microstructures in Al-9.8%Si-3ppmP cast alloy and Al-10.1%Si-4ppmP-108ppmSr cast alloy are shown in Figure 1. The microstructures of both alloys are almost the same in the two-dimensional image. It is difficult to distinguish them.

Specimens for nanotomography were cut from the cast ingot. Very small stick-shaped specimens with a section size of 50 μm × 50 μm and length of about 8 mm were manufactured by hand polishing. Five tensile specimens with 19.75 mm^2^ section × 30 mm length in a gauge part, which is a half size of JIS No.13 B (JIS Z 2241), were prepared from a position of 2 mm above from the bottom of the cast ingot, then the mechanical properties of the cast alloys were examined by a tensile testing machine (SHIMADZU AG-100 kNX, Kyoto, Japan).

**Figure 2:**
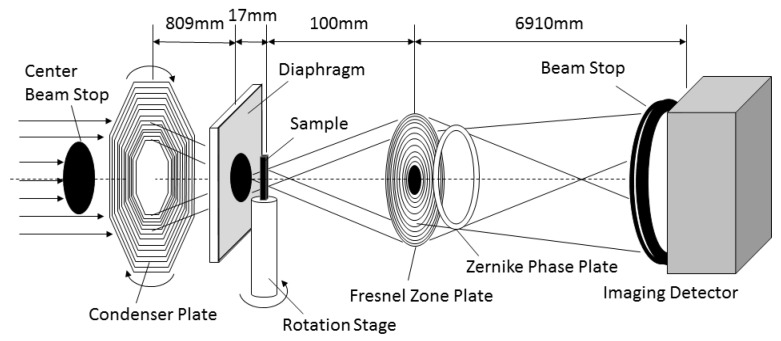
The schematic illustration of set-up of nanotomography in the experimental hutch.

**Figure 3:**
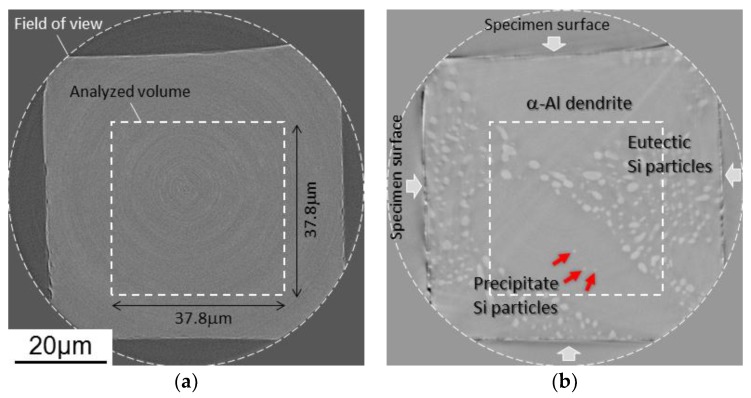
Slice images of nanotomography in Al-10.1%Si-4ppmP-108ppmSr cast alloy heat-treated at 773 K for 7.2 ks. The same slices are shown by (**a**) absorption contrast and (**b**) phase contrast. Field of view and analyze-volume position are indicated by white dashed-line circle and box. Eutectic Si-particles and precipitate Si-particles can be recognized as white objects in (**b**), though specimen surface only can be seen in (**a**).

Synchrotron radiation nanotomography was used for observation of three-dimensional morphology change in eutectic Si-particles during heat treatment. The synchrotron radiation experiment was performed at the undulator beam line of BL47XU in the Japanese synchrotron radiation facility, SPring-8 (Hyōgo, Japan). A schematic illustration of the nanotomography set-up in the experimental hutch is shown in Figure 2. X-ray energy of 8 keV, which was adjusted by a silicon (111) double-crystal monochromator (SPring-8 Standard Monochromator, sKohzu Precision Co.,Ltd, Kawasaki, Kanagawa, Japan), was selected for this observation. A Fresnel zone plate with an outermost zone width of 50 nm was installed as an X-ray objective of an imaging X-ray microscope (NTT-AT, Kawasaki, Kanagawa, Japan). A Zernike phase plate made from tantalum with a thickness of 0.96 μm was also installed at the back focal plane of the Fresnel zone plate. A two-dimensional image detector system consisting of a Gd_2_O_2_S:Tb scintillator, an optical relay lens and a complementary metal oxide semiconductor camera (Hamamatsu Photonics K.K., C11440-22C, Hamamatsu, Shizuoka, Japan) was used. Since the difference in atomic number between them is only one, there is little X-ray absorption contrast in the Al phase and Si phase as shown in Figure 3a. Therefore, Si-particles in the inside of the aluminum matrix were visualized by phase contrast using a Zernike phase plate as shown in Figure 3b. Exposure time of 250 ms was used and 1800 projections were captured during a 180° rotation, for tomography. Voxel size of (37.8 nm)^3^ was achieved in the reconstructed volume image in the set-up of this study.

Experimental procedure for the synchrotron radiation nanotomography observation was simple. The initial state of the sample, i.e., as-cast sample, was scanned. After the first tomography scan, the sample was heat-treated by taking it in and out of a compact air atmosphere furnace maintained at 773 K, and then was tomography scanned repeatedly at the same position at 450 s, 900 s, 1.8 ks, 3.6 ks, 7.2 ks and 14.4 ks. X-ray scanned data were reconstructed into a three-dimensional volume image by a conventional filtered convolution back-projection algorithm. A three-dimensional median filter (3 × 3 × 3) was applied to three-dimensional volume images reconstructed in order to reduce artifacts and image noise. Si-particles observed in the volume images were binarized and segmented with the thresholds value that were decided by comparing the obtained volume images to one another. The result of Si-particles segmentation was checked by visual inspection. Then if wrong connections existed among particles, such connections were carefully corrected one by one. Volume rendering software (VG studio Max 2.0, Volume Graphics, Heidelberg, Germany; and Amira 4.0, Thermo Fisher Scientific, Waltham, MA, USA) was used to visualize the three-dimensional morphology of Si-particles. The analyzed region for the morphology changes of eutectic Si-particles was extracted from a lower effect region of artifacts inside the sample. The analyzed regions were 56.7 μm × 28.4 μm × 27.2 μm and 37.8 μm × 37.8 μm × 41.8 μm in Al-9.8%Si-3ppmP sample and Al-10.1%Si-4ppmP-108ppmSr, respectively. Si-particles within the analyzed regions were segmented and labeled after binarization. Then, volume and surface area were measured for each of the labeled Si-particles.

## 3. Results

### 3.1. Mechanical Properties

**Figure 4:**
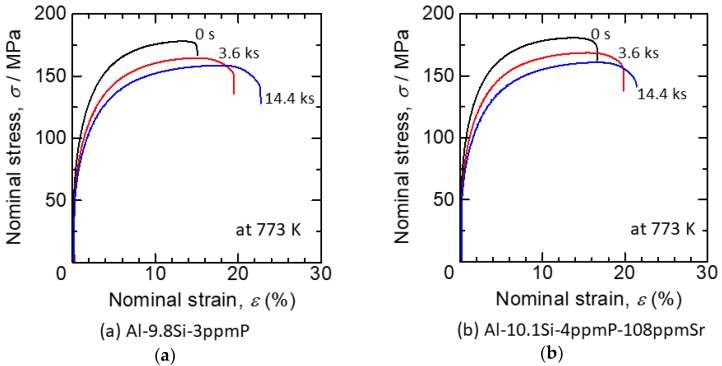
Stress-strain curves of tensile test in (**a**) Al-9.8%Si-3ppmP cast alloy and (**b**) Al-10.1%Si-4ppmP-108ppmSr cast alloy.

**Figure 5:**
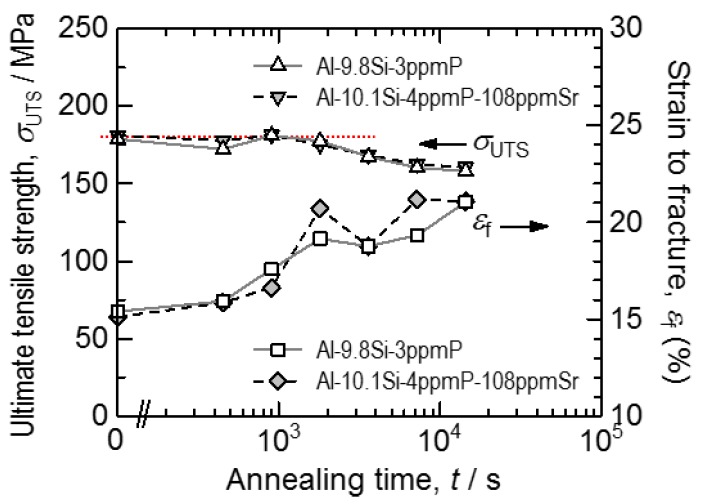
Changes in ultimate tensile strength and elongation during heat treatment at 773 K.

The stress-strain curves of a tensile test in Al-9.8%Si-3ppmP cast alloy and Al-10.1%Si-4ppmP-108ppmSr cast alloy are shown in Figure 4. The ultimate tensile strength decreases and elongation to failure increases in both samples with increasing heat treatment time. Both samples show almost a similar stress-strain relationship before and after heat treatment, though tensile strength in Al-10.1%Si-4ppmP-108ppmSr cast alloy is slightly higher than that in Al-9.8%Si-3ppmP cast alloy. In both alloys, heat treatment reduces yield stress and work hardening rate mildly. Elongations to both alloys are almost the same with the same heat treatment time. Note that Vickers hardness in Al-9.8%Si-3ppmP cast alloy and Al-10.1%Si-4ppmP-108ppmSr cast alloy were 58.5 HV and 59.6 HV, respectively. There was no difference in the dendrite secondary arm spacing (DASII)—which was approximately 37 μm—in both of the alloys. Changes in ultimate tensile strength and elongation (average of 5 specimens) are shown in Figure 5. By heat treatment, ultimate tensile strength decreases gradually and elongation increases in both alloys. The changes become particularly remarkable after 1.8 ks of heat treatment. With a short period of heat treatment, no differences are seen in either alloys. However, when applying heat treatment for a longer time, the mechanical properties in Al-10.1%Si-4ppmP-108ppmSr cast alloy become superior to that of Al-9.8%Si-3ppmP cast alloy.

### 3.2. Morphology Changes of Eutectic Si-Particles

**Figure 6:**
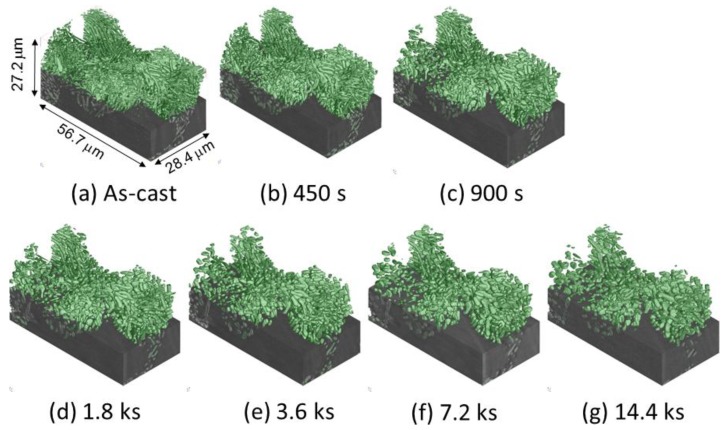
Three-dimensional volume images of eutectic Si-particles in Al-9.8%Si-3ppmP cast alloy. (**a**) As-cast, (**b**) heat-treated at 773 K for 450 s, (**c**) heat-treated at 773 K for 900 s, (**d**) heat-treated at 773 K for 1.8 ks, (**e**) heat-treated at 773 K for 3.6 ks, (**f**) heat-treated at 773 K for 7.2 ks, (**g**) heat-treated at 773 K for 14.4 ks.

Three-dimensional volume images of eutectic Si-particles in Al-9.8%Si-3ppmP cast alloy, which are obtained by synchrotron radiation nanotomography, are shown in Figure 6. Interior Si-particles are displayed removing the aluminum matrix in the top part of each figure. It can be confirmed that synchrotron radiation nanotomography is high resolution because the figure indicates the changes in a very small region with a size of 28.4 μm × 56.7 μm × 27.2 μm. In the as-cast state (heat treatment time, t = 0 s) as shown in Figure 6a, most of the eutectic Si-particles are of a straight rod-like shape, and a small plate-like shape is also seen partially. It is observed that the rod and plate-like Si-particles are connecting. Particle growth is confirmed during heat treatment up to 14.4 ks as shown in Figure 6b–g. The number of particles decrease gradually during particle growth. Although particle separation that makes particles segment into a small size is also observed, most of the particles maintain a high aspect ratio after 14.4 ks annealing.

**Figure 7:**
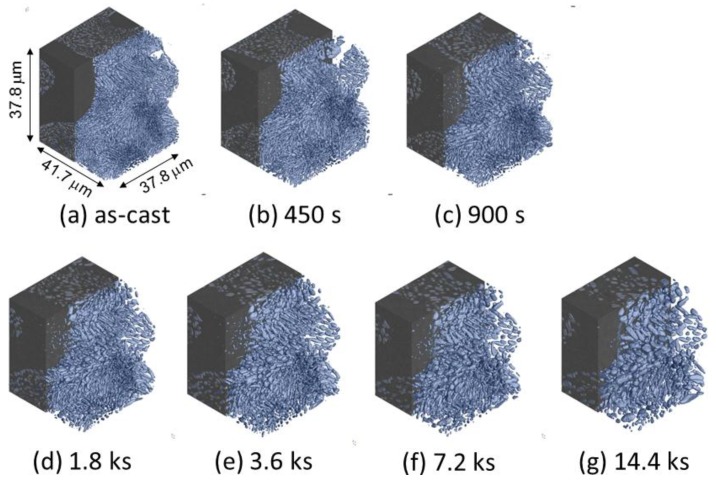
Three-dimensional volume images of eutectic Si-particles in Al-10.1%Si-4ppmP-108ppmSr cast alloy. (**a**) As-cast, (**b**) heat-treated at 773 K for 450 s, (**c**) heat-treated at 773 K for 900 s, (**d**) heat-treated at 773 K for 1.8 ks, (**e**) heat-treated at 773 K for 3.6 ks, (**f**) heat-treated at 773 K for 7.2 ks, (**g**) heat-treated at 773 K for 14.4 ks.

**Figure 8:**
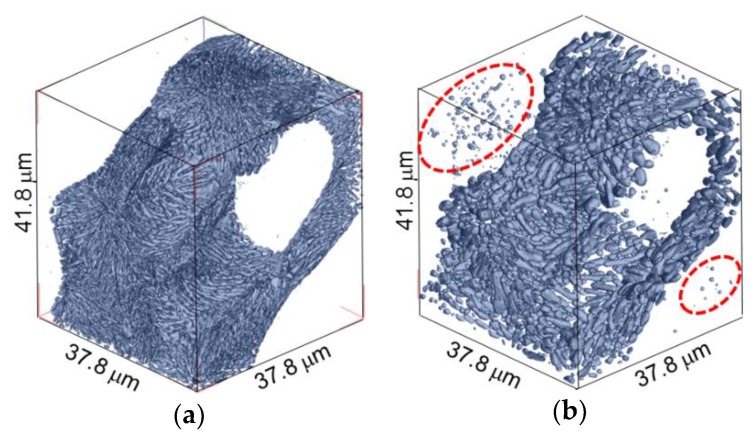
Three-dimensional volume images in Al-10.1%Si-4ppmP-108ppmSr cast alloy; (**a**) as-cast and (**b**) after 7.2 ks heat-treated. New Si-particle precipitate during heat treatment.

Figure 7 shows a three-dimensional volume image of Al-10.1%Si-4ppmP-108ppmSr cast alloy. In the as-cast state (Figure 7a), fine rod-like Si-particles are observed similar to Al-9.8%Si-3ppmP cast alloy. However, the entire morphology of Si-particles in Sr-modified alloy are that of a coral-like shape with multiple branches. Particle size is slightly finer than that in Al-9.8%Si-3ppmP cast alloy. The Si-particles grow gradually with fragmentation during heat treatment, and spheroidize after 14.4 ks. In Al-10.1%Si-4ppmP-108ppmSr cast alloy, formation of Sr precipitations was confirmed in primary α-Al dendrite during heat treatment. Three-dimensional volume images in (a) as-cast and (b) after 7.2 ks heat-treated are shown in Figure 8. New Si-particles, which are not found in the as-cast state, are observed in the outside region of eutectic phase in which Si-particles are gathering. The presence of precipitate Si-particles is also confirmed in the slice image shown in Figure 3b. It can be concluded that the particles are not Sr compounds but Si because the particles have disappeared in the absorption images shown in Figure 3a.

**Figure 9:**
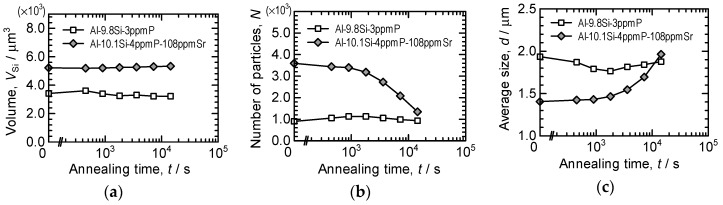
Changes in total Si-particle volume, number of Si-particle and average Si-particle size during heat treatment. Initial total Si-particle volume was 3416.645 μm^3^ and 5217.278 μm^3^ in Al-9.8%Si-3ppmP cast alloy and Al-10.1%Si-4ppmP-108ppmSr cast alloy, respectively. (**a**) total Si-particle volume, (**b**) number of Si-particles and (**c**) average Si-particle size.

Figure 9 indicates changes in total Si-particle volume, number of Si-particles and average Si-particle size (sphere-equivalent diameter) during heat treatment. These statistics were obtained from the microstructures shown in Figure 6 and Figure 7 by three-dimensional image processing analysis. The total Si-particle volume in Al-10.1%Si-4ppmP-108ppmSr cast alloy is larger than that in Al-9.8%Si-3ppmP cast alloy. Volume fraction of Si phase in Al-10%Si alloy should be approximately 11.4%. However, the volume fractions were slightly small in the volumes analyzed and were 7.8% and 8.7% in Al-9.8%Si-3ppmP cast alloy and Al-10.1%Si-4ppmP-108ppmSr cast alloy, respectively. This is due to inhomogeneities of microstructure and the small field of view size in nanotomography. The amount of Si content does not differ in both alloys. The total Si-particle volume in Al-9.8%Si-3ppmP cast alloy looks to slightly decrease during heat treatment. This change is due to particles on the edge of view. Total Si-particle volume is almost constant during heat treatment in both alloys. In the as-cast, the number of Si-particles in Al-10.1%Si-4ppmP-108ppmSr cast alloy is larger than that in Al-9.8%Si-3ppmP cast alloy. This is because total Si-particle volume is large in Al-10.1%Si-4ppmP-108ppmSr cast alloy, and the particle size is small as observed in Figure 7. The number of particles decreases in both alloys during heat treatment. In Al-9.8%Si-3ppmP cast alloy, fragmentation of Si-particles, which is observed in the early stage of heat treatment, causes an increase of number of particles temporarily. As shown in Figure 8, Si-particles precipitate into α-Al dendrites during heat treatment. However, the precipitation has no effect on the number of Si-particles, because the number increase is small compared with the number decrease by particle growth. In Al-9.8%Si-3ppmP cast alloy, average Si-particle size decreases, and then increases. This decrease at the early stage is also due to fragmentation of Si-particles. In case of Al-10.1%Si-4ppmP-108ppmSr cast alloy, a little increase of Si-particle size is found in the early stage of heat treatment, and then the size increases rapidly in the later period of heat treatment.

## 4. Discussion

Looking at Figure 9, the situations in which particles grow while keeping volume constant are Ostwald ripening. According to classical particle growth theory, a growth of Ostwald ripening [31] is formulated as

**Figure 10:**
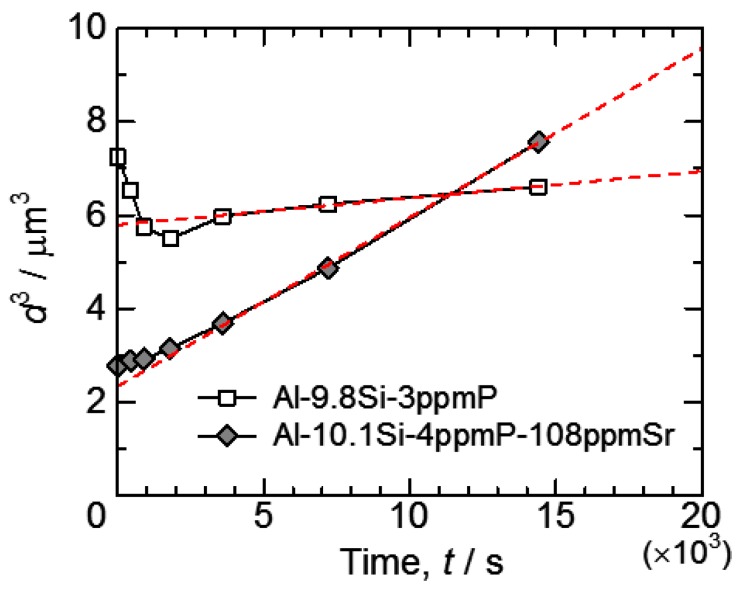
The relationship between cube of average particle size and annealing time.

*d*^3^ − *d*_0_^3^ = *kt* (1)

in a diffusion control situation. Here, *d* and *d*_0_ are the average particle diameter and initial average particle diameter, *k* is constant and *t* is annealing time. The relationship between the cube of average particle diameter and annealing time in this study is shown in Figure 10. The early stage of particle growth in Al-9.8%Si-3ppmP cast alloy does not correspond to the growth manner expressed in Equation (1). The Si-particles grow proportionally after 3.6 ks of heat treatment. The particle growth in Al-10.1%Si-4ppmP-108ppmSr cast alloy almost obeys Equation (1) though a little difference is seen in the early stage of growth. It is found that Si-particle growth in Al-10.1%Si-4ppmP-108ppmSr cast alloy is faster than that in Al-9.8%Si-3ppmP cast alloy. A very small difference in particle growth rate had been expected because the alloys were simple binary Al-Si system alloys though there was a difference in 108ppm Sr content. However, a six-times difference is recognized in the comparison with the slopes of fitting lines between both of the alloys in the later stage of growth.

**Figure 11:**
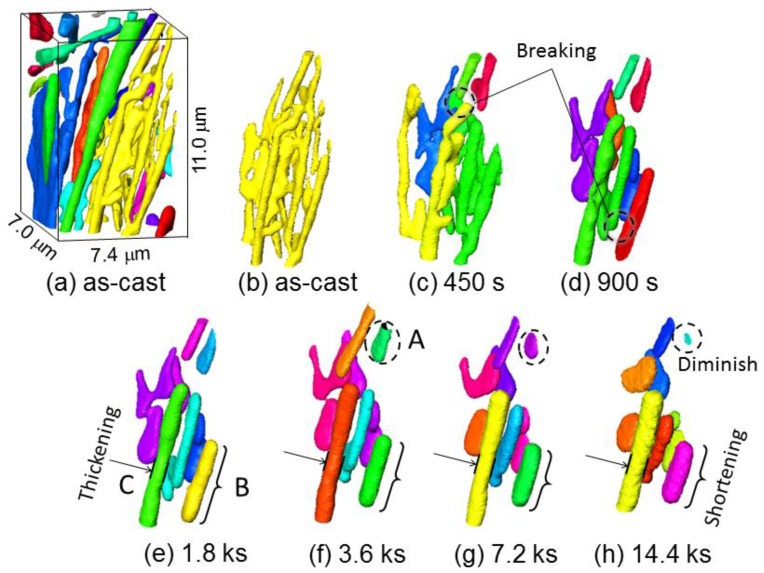
Magnification of three-dimensional volume image, illustrating morphology changes of Si-particles in Al-9.8%Si-3ppmP cast alloy. (**a**) as-cast, (**b**) as-cast (one particle), (**c**) heat-treated at 773 K for 450 s, (**d**) heat-treated at 773 K for 900 s. (**e**) heat-treated at 773 K for 1.8 ks. (**f**) heat-treated at 773 K for 3.6 ks, (**g**) heat-treated at 773 K for 7.2 ks, (**h**) heat-treated at 773 K for 14.4 ks.

By magnifying a three-dimensional volume image, morphology changes of Si-particles in Al-9.8%Si-3ppmP cast alloy are shown in Figure 11. Actually, Si-particles distribute densely as shown in Figure 11a. To understand the morphological changes of particles easily, an image removing surrounding particles is Figure 11b. In the as-cast, the morphology of Si-particles is mildly complex, possessing a fine rod-like shape, which is elongated along the solidification direction, and a partial small plate-like shape, which is broader than the rod part. While heat treatment is progressing, the Si-particles are divided into plural segments (Figure 11c,d). After that, fragmented particles become gradually round and approach into a sphere-like shape that has the smallest surface area (Particle A in Figure 11e). An elongated Particle B shown in Figure 11e gradually shortens in length, and then becomes close to a sphere-like shape. An elongated Particle C seen in the center of the figures thickens in diameter during heat treatment. However, the tip position does not change so much after 1.8 ks of heat treatment and the small change is observed in particle shortening along longitudinal direction. It is found that the morphology change is slightly different depending on the length of the rod-like shaped particles.

**Figure 12:**
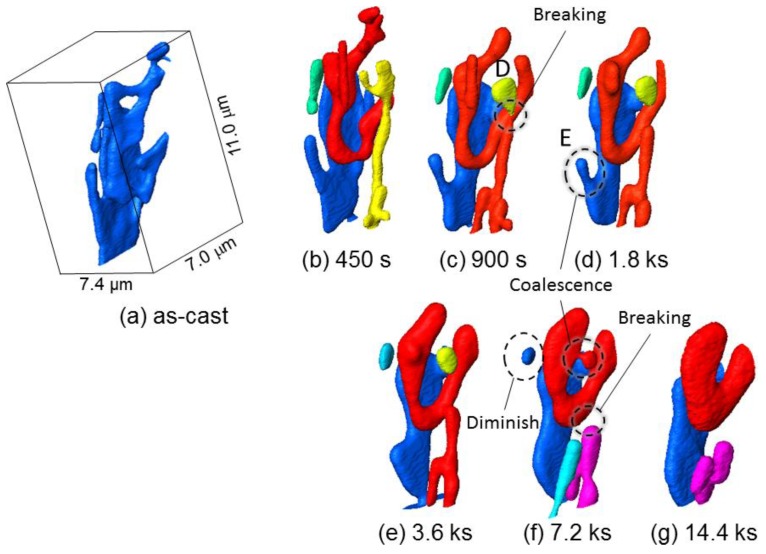
Three-dimensional images viewing Figure 11 from the back side. (**a**) as-cast (one particle), (**b**) heat-treated at 773 K for 450 s, (**c**) heat-treated at 773 K for 900 ks, (**d**) heat-treated at 773 K for 1.8 ks, (**e**) heat-treated at 773 K for 3.6 ks, (**f**) heat-treated at 773 K for 7.2 ks, (**g**) heat-treated at 773 K for 14.4 ks.

Figure 12 shows three-dimensional images viewing Figure 11 from the rear side. Particle D fragmented at 900 s heat treatment seen in Figure 12c becomes a sphere-like shape in (d) 1.8 ks and (f) 3.6 ks of heat treatment. The particle is merged with a U-shape particle growing behind its back. The U-shape particle that absorbed Particle D probably will close a gap and be a sphere-shaped particle if heat treatment continues furthermore. A protuberance indicated by the letter E in Figure 12d is not cut off at the neck, shortens in length gradually and finally is absorbed by a plate-shape particle. The morphology change of Si-particles is very complex in Al-9.8%Si-3ppmP cast alloy. It was found that the changes during heat treatment were not only just fragmentation and spheroidizing.

**Figure 13:**
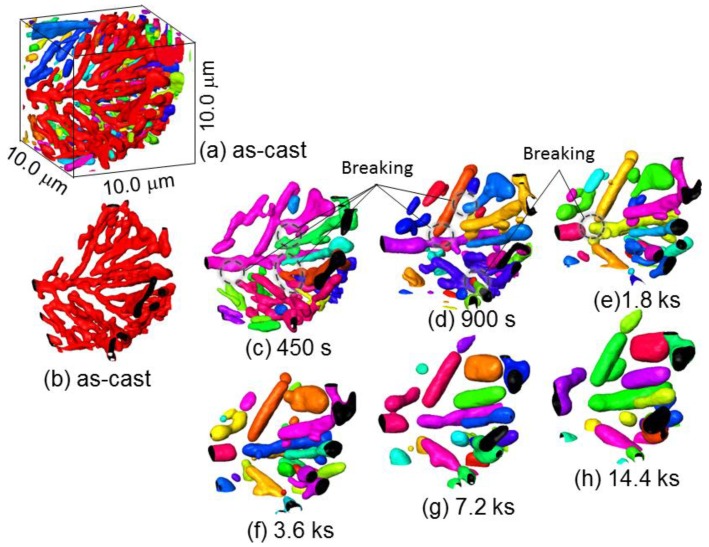
Magnified three-dimensional images of Si-particles in Al-10.1%Si-4ppmP-108ppmSr cast alloy. (**a**) as-cast, (**b**) as-cast (one particle), (**c**) heat-treated at 773 K for 450 ks, (**d**) heat-treated at 773 K for 900 ks, (**e**) heat-treated at 773 K for 1.8 ks, (**f**) heat-treated at 773 K for 3.6 ks, (**g**) heat-treated at 773 K for 7.2 ks, (**h**) heat-treated at 773 K for 14.4 ks.

Figure 13 shows magnified three-dimensional images of Si-particles in Al-10.1%Si-4ppmP-108ppmSr cast alloy. Figure 13a shows the as-cast state displaying peripheral particles. One particle is shown in Figure 13b by removing the peripheral particles. It can be confirmed that Si-particles in Al-10.1%Si-4ppmP-108ppmSr cast alloy are coral-like complex shapes having numerous branches. As well as Al-9.8%Si-3ppmP cast alloy, separation of Si-particles is found at branches and necks surrounded by dashed lines shown in Figure 13c–e. Frequency of separation in Al-10.1%Si-4ppmP-108ppmSr cast alloy is higher than that in Al-9.8%Si-3ppmP cast alloy because the Si-particles in Al-10.1%Si-4ppmP-108ppmSr cast alloy possess many branches in as-cast. Si-particle segmentations formed by separation thicken gradually. Then, the shape approaches a sphere-like form, with a shortening the length of longitudinal direction. Separation also causes a long trunk of Si-particles, as seen in the center of the figure. The trunk becomes segmented and grows to a sphere-like shape. Such behaviors correspond to those that we had expected. However, the morphology change was clearly different with the observed elongated particles in Al-9.8%Si-3ppmP cast alloy. Therefore, the difference of growth rates in the two cast alloys would be brought about by the difference of growth behavior at long elongated Si-particles. That is, the slow growth rate in Al-9.8%Si-3ppmP cast alloy in heat treatment is due to less separation of long elongated rod-shape particles, which are the main morphological features of eutectic Si-particles by the solidification of Al-9.8%Si-3ppmP cast alloy. In addition, the solidification reaction is different in the two cast alloys as seen in the different eutectic Si-particle morphology. Not only is the eutectic Si-particle size in Al-10.1%Si-4ppmP-108ppmSr cast alloy smaller than that in Al-9.8%Si-3ppmP cast alloy, but the eutectic grain formed in the eutectic reaction is also of a fine size in Al-10.1%Si-4ppmP-108ppmSr cast alloy. Therefore, the contribution of grain boundary diffusion in addition to lattice diffusion could also be a factor.

As results of the detail three-dimensional observation of microstructural change through Figure 11 and Figure 13, it was observed that many Si-particles separate and isolate as small segments in the early stage of heat treatment up to 900 s. Considering the relationship between microstructure and mechanical property, it is found that the mechanical properties shown in Figure 5 also start to change after 900 s heat treatment. The non-destructive observation in this study by means of synchrotron radiation nanotomography supports the idea that the connectivity of the strengthening phase affects strength and elongation as reported recently [32,33]. Further investigation and consideration for this will be possible in image-based simulations that are constructed from three-dimensional volume image tomography.

## 5. Conclusions

In this study, a series of three-dimensional morphology changes of fine eutectic Si-particles during heat treatment have been investigated in Self-modified and Sr-modified Al-10%Si cast alloys by means of synchrotron radiation nanotomography using a Fresnel zone plate and a Zernike phase plate. The morphology of eutectic Si-particles was rod-like in shape in the self-modified sample of Al-9.8%Si-3ppmP cast alloy. In the Sr-modified sample of Al-10.1%Si-4ppmP-108ppmSr cast alloy, Si-particle was a coral-like shape in the as-cast. The coral-like shape particles observed in Al-10.1%Si-4ppmP-108ppmSr cast alloy fragmented at branch and neck during heat treatment at 773 K. The fragmentation occurred up to 900 s. After that, the fragmented particles grew and spheroidized by Ostwald ripening. The rate of Ostwald ripening in Al-10.1%Si-4ppmP-108ppmSr cast alloy was faster than that in Al-9.8%Si-3ppmP cast alloy. On the other hand, rod-like shaped eutectic Si-particles observed in Al-9.8%Si-3ppmP cast alloy were larger in size compared to the particle size in Al-10.1%Si-4ppmP-108ppmSr cast alloy. In Al-9.8%Si-3ppmP cast alloy, separation of eutectic Si-particles occurred up to approximately 900 s, which was similar tendency to that in Al-10.1%Si-4ppmP-108ppmSr cast alloy. The frequency of separation was low due not to the coral-like shape but the rod-like shape. Three-dimensional morphology changes of fine eutectic Si-particles in both cast alloys, specifically fragmentation and spheroidizing, can be connected to changes in mechanical properties. In the rod-like shape of Si-particles obtained in a self-modified sample of Al-9.8%Si-3ppmP cast alloy, however, it was found that the morphology change behavior was very complex. By non-destructive continuous observation using nanotomography, it was revealed that relatively long rod-shape particles grew slowly without separation. It is also observed that a protuberance was absorbed into a small plate-shape part. Moreover, very complex behavior was observed where a rod-shape particle separated at the neck, spheroidized and was then absorbed by a neighboring larger particle.

## Figures and Tables

**Table 1:** Chemical composition of prepared cast alloys (wt.%).

Sample	Si	P	Sr	Cu	Al
Self-modified alloy	9.8	0.0003	<0.00001	0.08	Bal.
Sr-modified alloy	10.1	0.0004	0.0108	0.07	Bal.
